# Supplementary material for: Identification and characterization of N9-methyltransferase involved in converting caffeine into non-stimulatory theacrine in tea
Source: Nat Commun. 2020 Mar 19;11:1473. doi: 10.1038/s41467-020-15324-7 (PMC7081346; doi:10.1038/s41467-020-15324-7)
Supplement: Supplementary file 1 — Supplementary Information [file 41467_2020_15324_MOESM1_ESM.pdf]

Supplementary Information

Identification and characterization of *N*9-methyltransferase involved in converting caffeine into non-stimulatory theacrine in tea

Zhang et al.

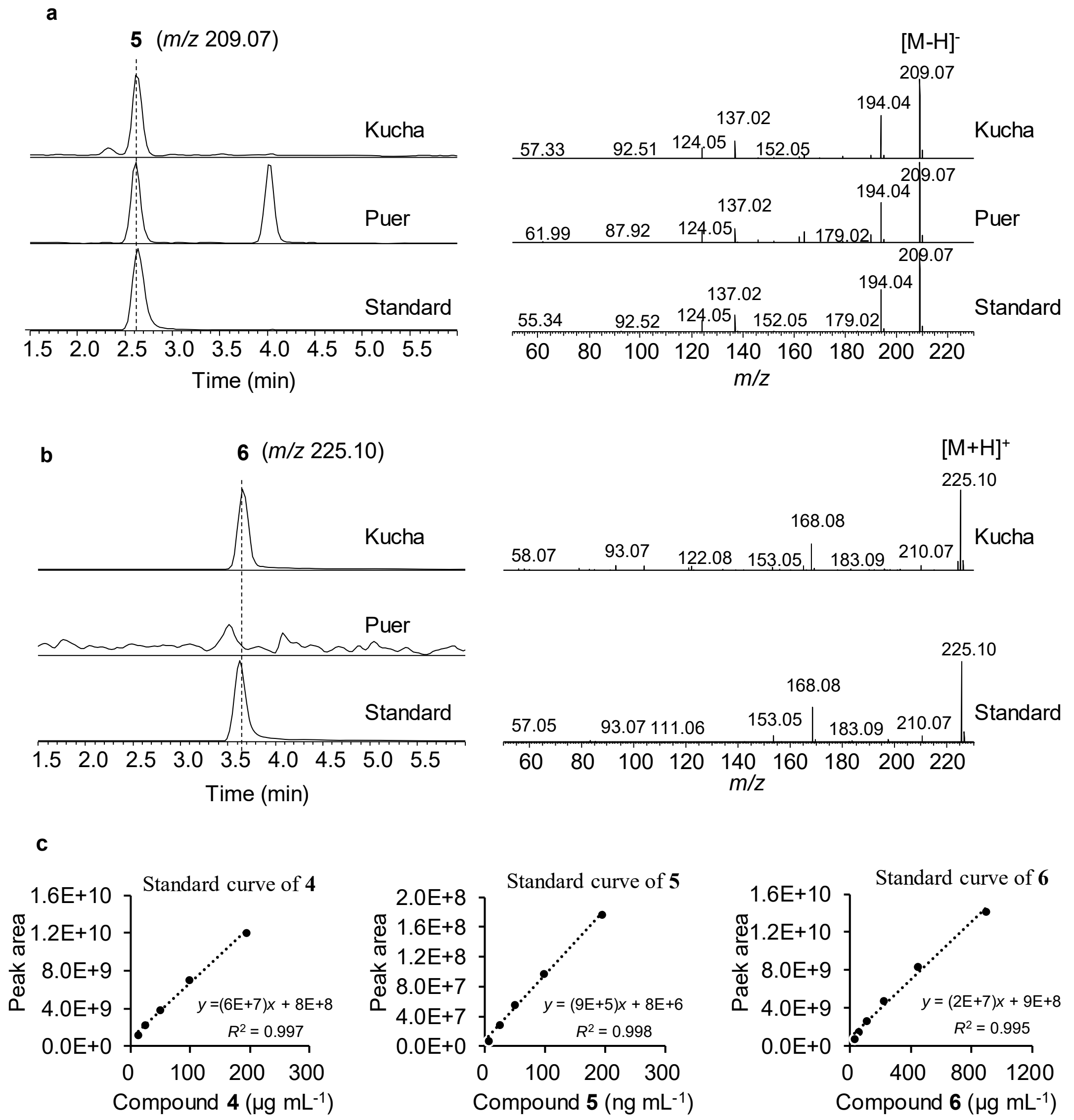

Supplementary Figure 1. Qualitative and quantitative analysis of major xanthine alkaloids in Puer and Kucha. (**a**) Identification of 1,3,7-trimethyluric acid (**5**) in tea plants. Left panel: extract ion current of *m*/*z* 209.07 in Kucha, Puer and standard substance; Right panel: MS/MS spectrum of *m*/*z* 209.07 in Kucha, Puer and standard substance. (**b**) Identification of theacrine (**6**) in tea plants. Left panel: extract ion current of *m*/*z* 225.10 in Kucha, Puer and standard substance; Right panel: MS/MS spectrum of *m*/*z* 225.10 in Kucha and standard substance. (**c**) Standard curve of concentration and

peak area of xanthine alkaloids.

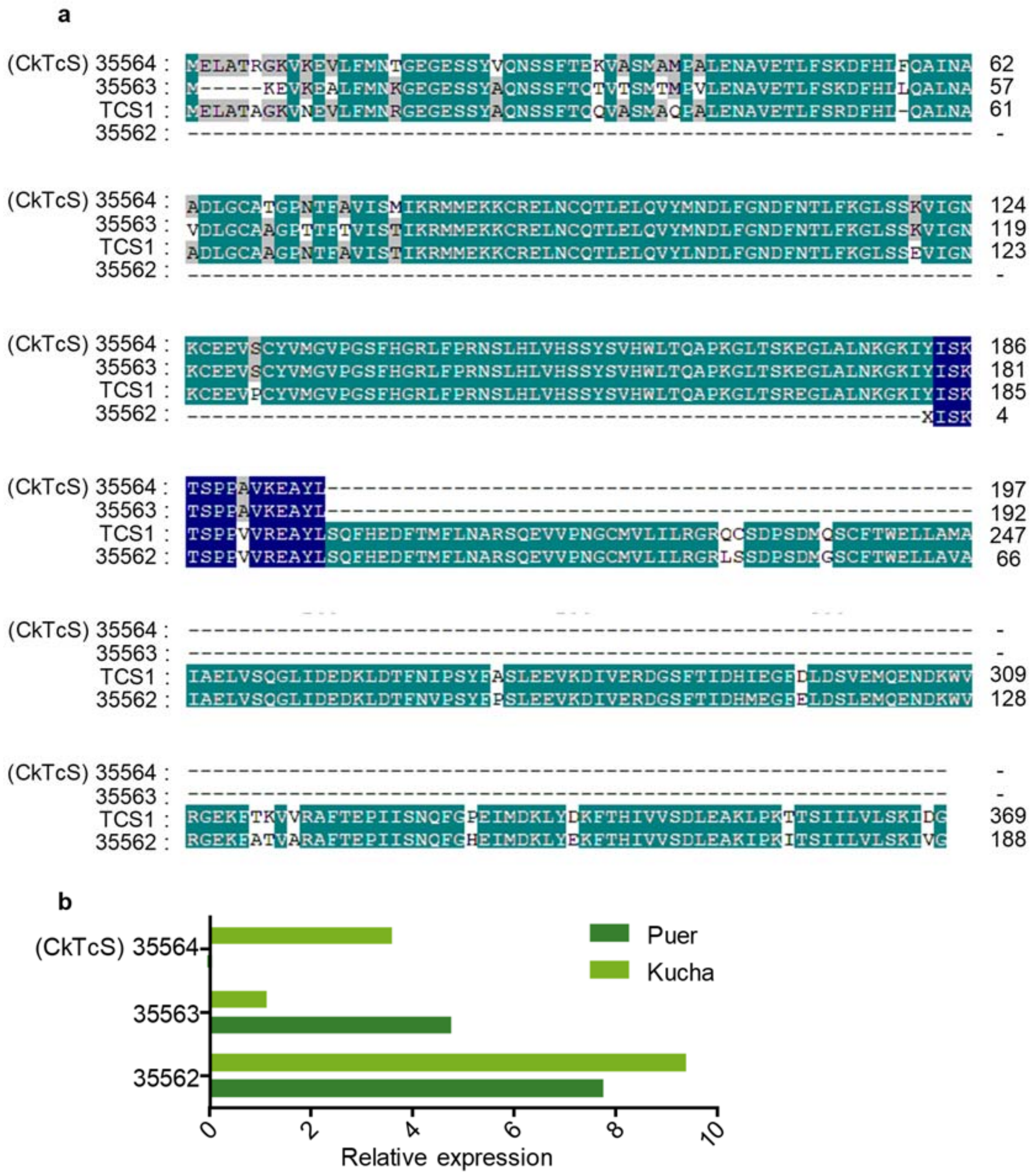

Supplementary Figure 2. Transcriptome data analysis (**a**) Amino acid sequences alignment between TCS1 and the *N*-methyltransferase sequences obtained from RNA-seq. The shading regions indicates the conserved amino acids among these NMTs. (**b**) Differential expression analysis of 35562, 35563 and 35564 between Puer and Kucha.

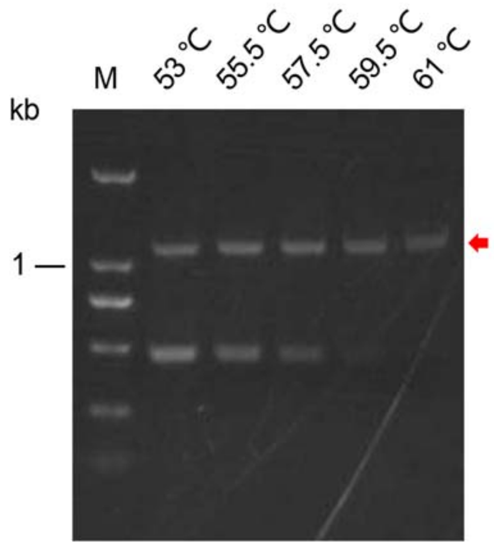

Supplementary Figure 3. Cloning of *N*-methyltransferase genes from Kucha. Total RNA was prepared from Kucha leaves and reverse transcripted into cDNA. The *N*-methyltransferase genes were then amplified by PCR using cDNA as template at different annealing temperatures. The target PCR bands were indicated by red arrow.

a

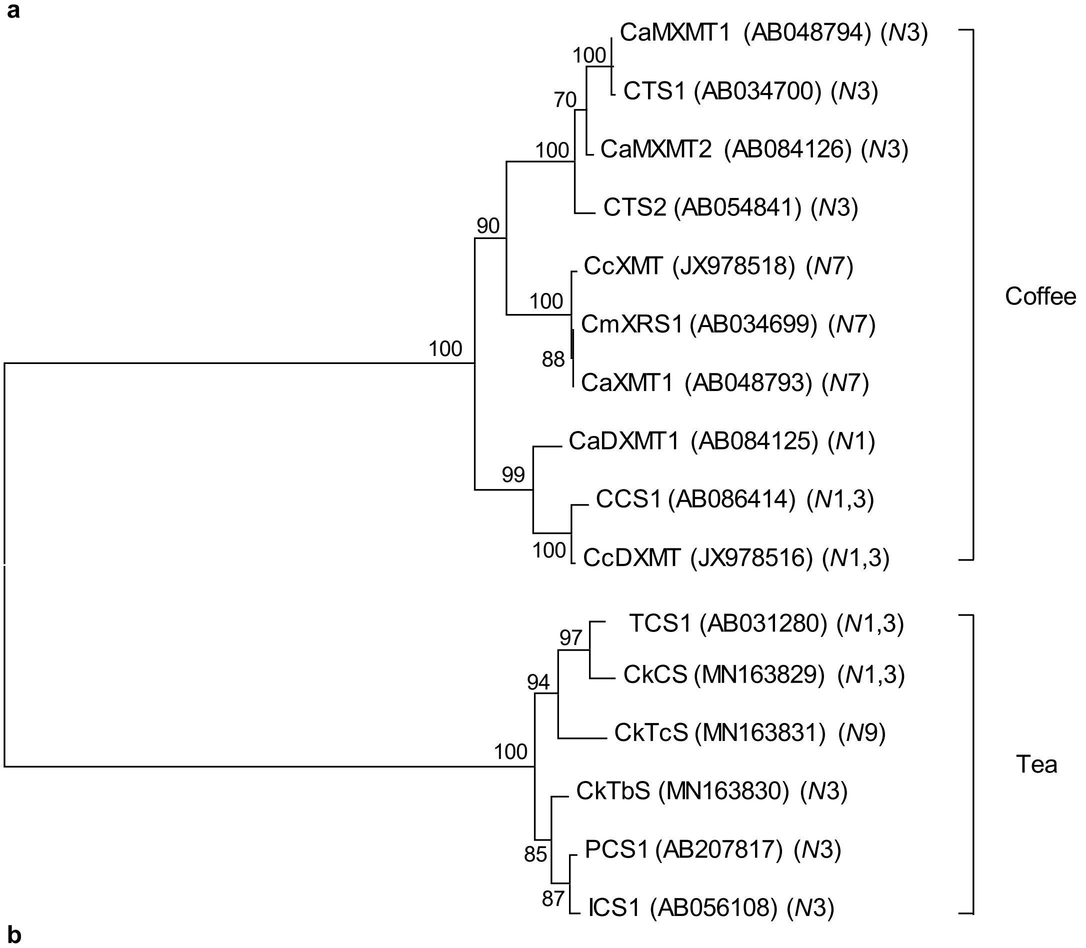

b

	TCS1	CkCS	CkTcS	CkTbS	PCS1	ICS1
TCS1	100.0	96.8	92.4	91.4	90.9	92.0
CkCS	96.8	100.0	91.6	90.3	89.8	91.2
CkTcS	92.4	91.6	100.0	92.2	90.9	92.0
CkTbS	91.4	90.3	92.2	100.0	95.9	97.3
PCS1	90.9	89.8	90.9	95.9	100.0	98.6
ICS1	92.0	91.2	92.0	97.3	98.6	100.0

Supplementary Figure 4. A phylogenetic tree of amino acid sequences of *N*-methyltransferases from tea and coffee plants. (a) Phylogenetic analysis of the amino acid sequences for TCS1 (*Camellia sinensis*), ICS1 (*Camellia irrawadiensis*), PCS1 (*Camellia ptilophylla*), CkCS (*Camellia Kucha*), CkTbS (*Camellia Kucha*) and CkTcS (*Camellia Kucha*) from tea plants., and those for CaMXMT1 (*Coffea arabica*), CmXRS1 (*Coffea arabica*), CCS1 (*Coffea arabica*), CTS1 (*Coffea arabica*), CTS2 (*Coffea arabica*), CaXMT1 (*Coffea arabica*), CaMXMT2 (*Coffea arabica*),

CaDXMT1 (*Coffea arabica*), CcXMT (*Coffea canephora*) and CcDXMT (*Coffea canephora*) from coffee plants. The tree was constructed by MEGA 6.0. with the maximum likelihood method. The numbers on the nodes of phylogenetic tree indicate bootstrap values, which represent the phylogenetic confidence of the tree topology. The functional activities of *N*-methyltransferases are indicated as *N*7: *N*7-methyltransferase activity, *N*1,3: *N*1,3-methyltransferase activity, *N*3: *N*3-methyltransferase activity, *N*1: *N*1-methyltransferase activity, *N*9: *N*9-methyltransferase activity. (b) A similarity matrix of the amino acid identity between the *N*-methyltransferases from tea.

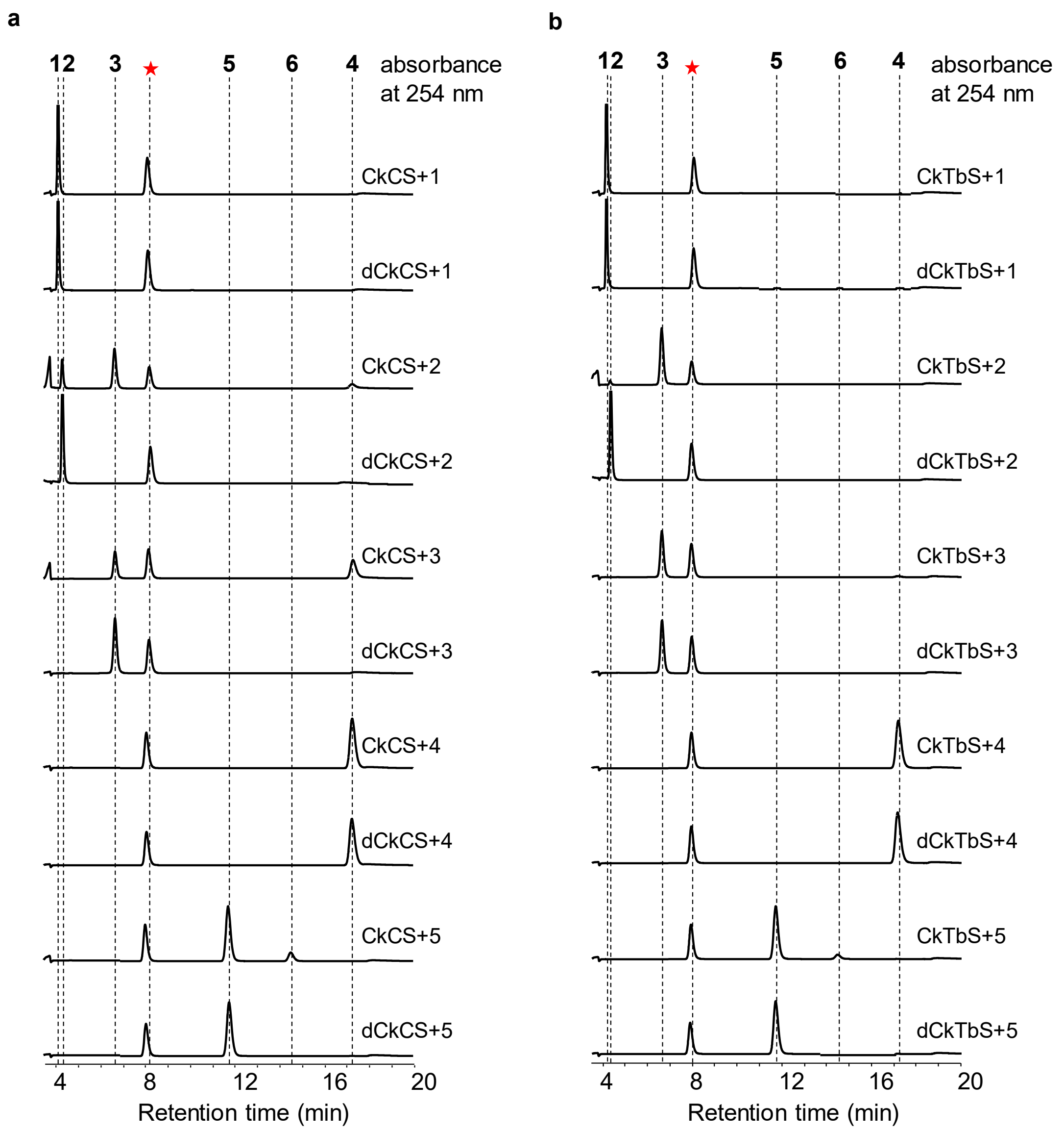

Supplementary Figure 5. *In vitro N*-methyltransferase activity of CkCS and CkTbS recombinant proteins. Compounds **1-6** represent xanthosine, 7-methylxanthine, theobromine, caffeine, 1,3,7-trimethyluric acid and theacrine, respectively. (**a**) HPLC analysis of *in vitro* reaction products of CkCS and denatured CkCS (dCkCS) with **1**, **2**, **3**, **4**, and **5**. (**b**) HPLC analysis of *in vitro* reaction products of CkTbS and denatured CkTbS (dCkTbS) with **1**, **2**, **3**, **4** and **5**. The absorbance wavelength was set at 254 nm. In all HPLC chromatograms, red asterisks indicate impurity compound from the SAM reagent.

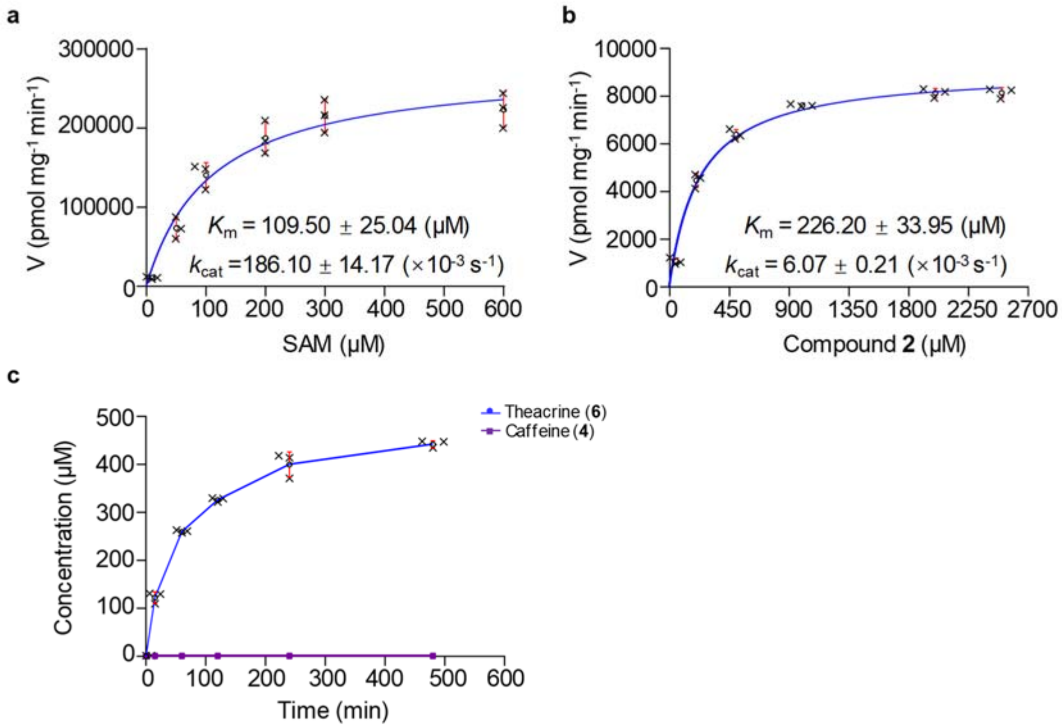

Supplementary Figure 6. Steady state kinetic analysis of CkTcS with SAM or 2 and comparison of the methylation activity of CkTcS towards 3 and 5. Kinetic parameters for CkTcS with SAM at a saturation concentration of 50 μM **5** (**a**) and CkTcS with **2** at a saturation concentration of 1.5 mM SAM (**b**). Initial velocities are shown as cycles and indicated as mean ± SD (n = 3). The blue line represents the nonlinear least-squares fit of the initial velocities versus substrate concentrations to the hyperbolic Michaelis-Menten equation. The corresponding dot plots are overlaid on the figure. (**c**) Comparison of the methylation activity of CkTcS towards **3** and **5** via a time course analysis. The reaction products caffeine (**4**) and theacrine (**6**) were quantified. The data represents mean ± SD (n = 3). The corresponding dot plots are overlaid on the figure.

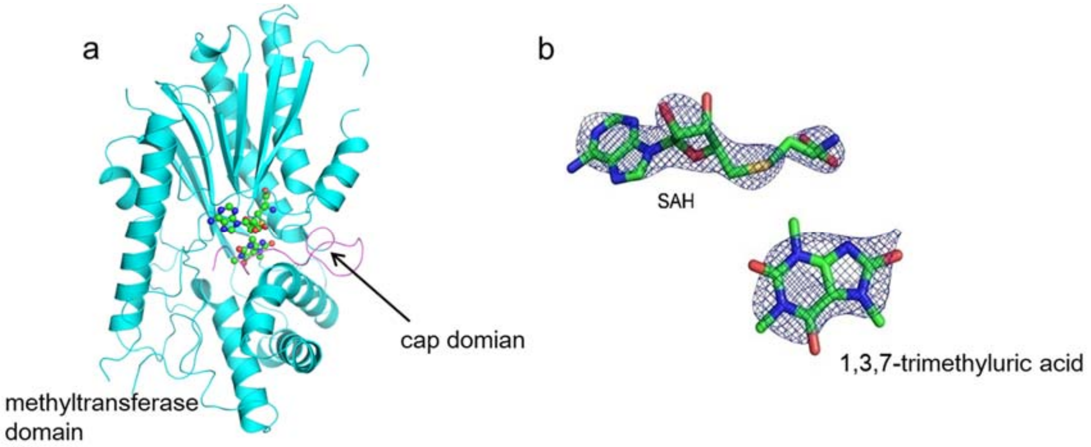

Supplementary Figure 7. The structure of CkTcS monomer. (**a**) Crystal structure of a monomer of the CkTcS-SAH-1,3,7-trimethyluric acid complex. CkTcS consists of an *N*-terminal cap domain (red) and a methyltransferase domain (Cyan). SAH and 1,3,7-trimethyluric acid are shown in stick representation. (**b**) 2Fo-Fc omit map of SAH and 1,3,7-trimethyluric acid in the CkTcS-SAH-1,3,7-trimethyluric acid complex structure at a contour level of 1.5 σ .

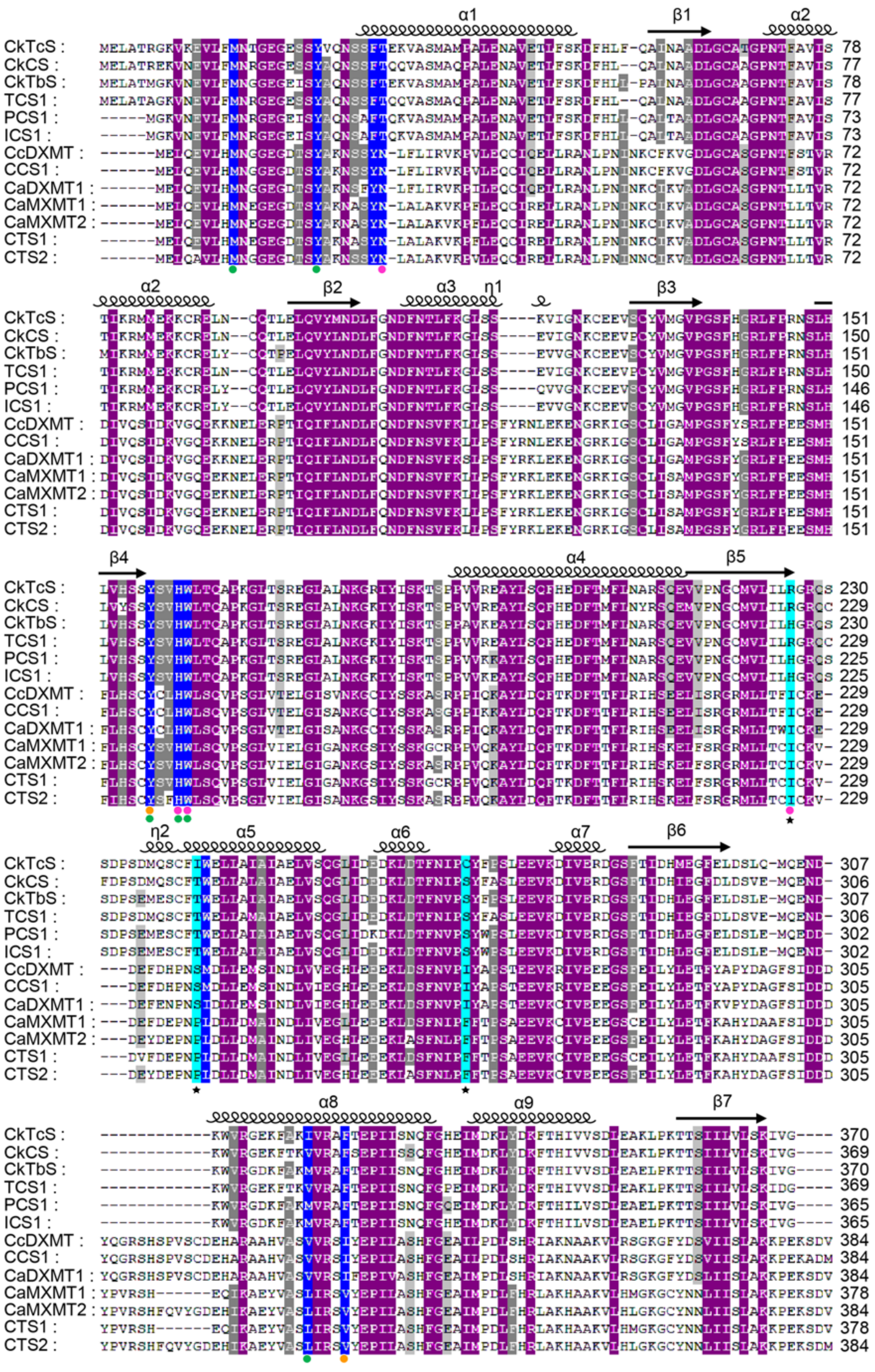

Supplementary Figure 8. Sequence alignment of *N*-methyltransferase. Secondary

structures of CkTcS (an *N*-methyltransferase with identical sequence was also cloned from Puer) are shown as black helices and arrows on top of the sequence. Residues lining the 1,3,7-trimethyluric acid binding pocket are highlighted by the dark blue box (conserved residues) and the bright blue box (variable residues). Residues forming direct hydrogen bond with 1,3,7-trimethyluric acid are labeled with pink dots under the sequence, and residues involved in π-π-stacking with orange dots. Conserved residues between *N*-methyltransferases are represented by grass green dots. The residues exchanged in Figure 4e) are highlighted by black star.

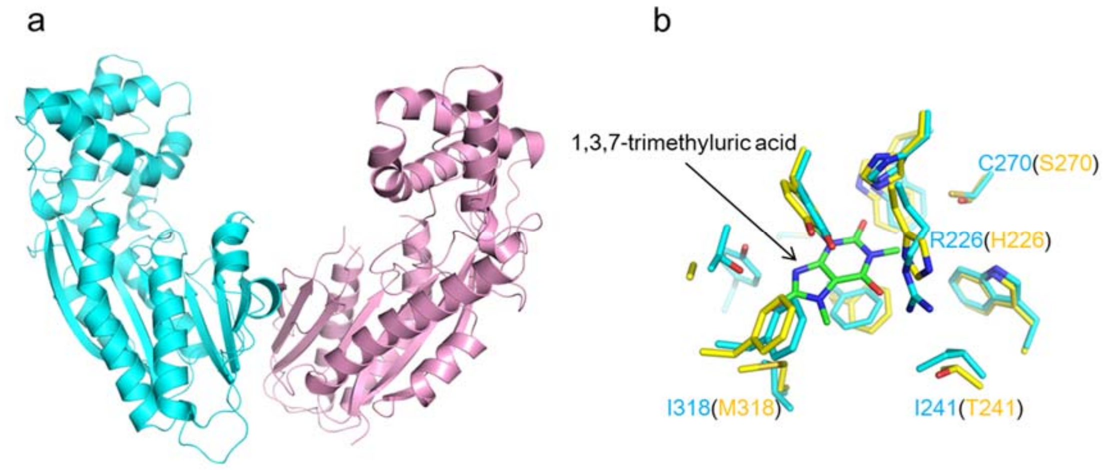

Supplementary Figure 9. Crystal structure of CkTbS. (**a**) Crystal structure of the CkTbS dimer. (**b**) Overlap of the substrate binding pockets on CkTcS (Cyan) and CkTbS (yellow). Four variable residues are labeled.

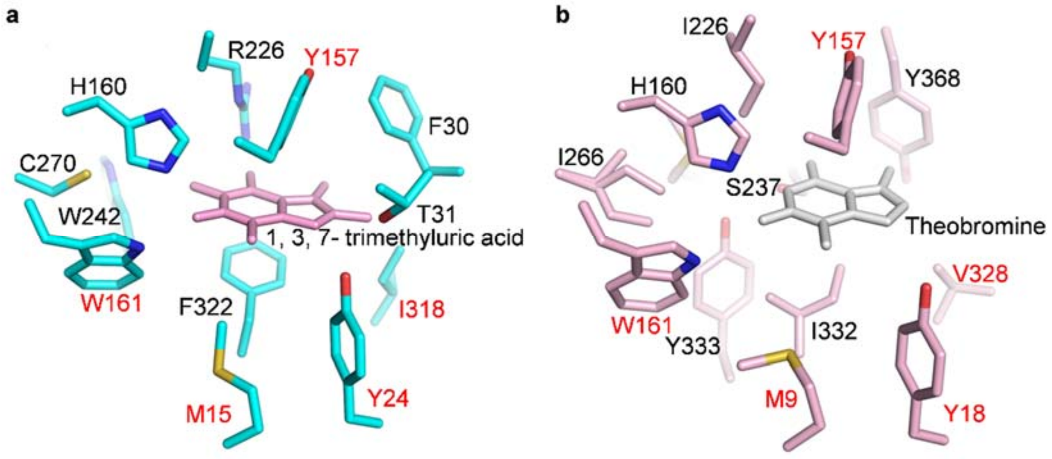

Supplementary Figure 10. Structural comparison of the substrate binding pockets of CkTcS and DXMT. Residues lining the CkTcS (**a**) and DXMT (**b**) are labeled. The sequence- and function-conserved residues are labeled in red. The substrate 1,3,7-trimethyluric acid and theobromine are shown as pink and grey sticks, respectively. There are two theobromine molecules in the DXMT structure, only the one that adopts the same orientation as that of 1,3,7-trimethyluric acid in CkTcS was exhibited.

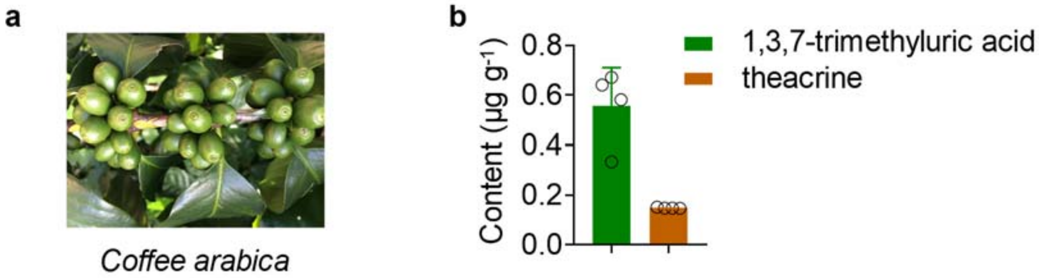

Supplementary Figure 11. Measurement of 1,3,7-trimethyluric acid and theacrine in coffee plant. (**a**) A picture of the coffee beans of *coffee arabica*. (**b**) The content of 1,3,7-trimethyluric acid and theacrine in the beans of *coffee arabica*. The data represents mean ± SD (n = 4). The corresponding dot plots are overlaid on the figure.

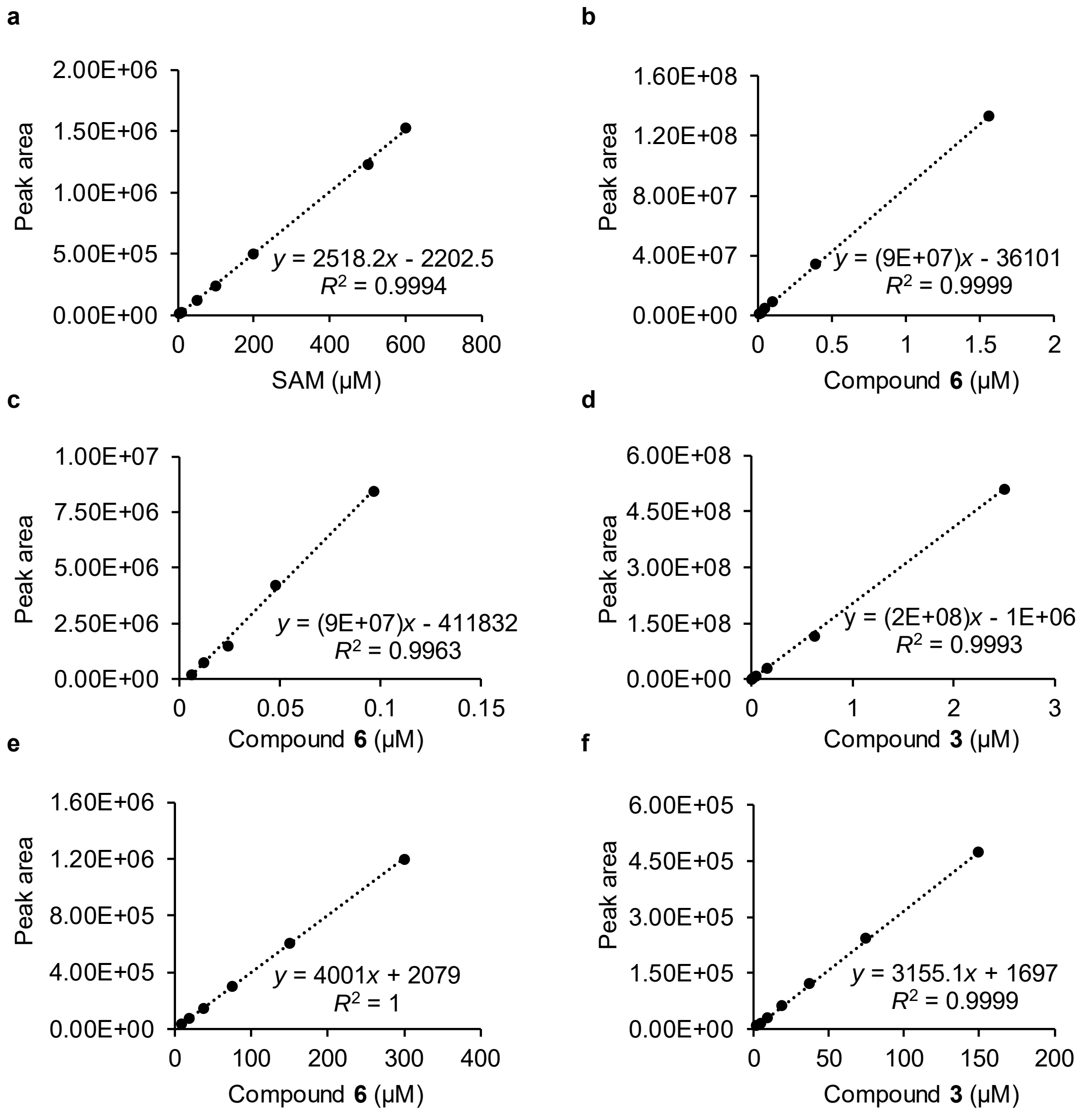

Supplementary Figure 12. Quantitative analysis of different substrate using HPLC-UV or HPLC-MS. (**a**) Standard curve of SAM using HPLC-UV. (**b**) Standard curve of compound **6** to determine the kinetic parameters of CkTcS and CkTbS with **5** using HPLC-MS. (**c**) Standard curve of compound **6** to determine the kinetic parameters of CkCS with **5** using HPLC-MS. (**d**) Standard curve of compound **3** using HPLC-MS. (**e**) Standard curve of compound **6** using HPLC-UV. (**f**) Standard curve of compound **3** using HPLC-UV.

Supplementary Table 1. Enzyme Kinetics of CkTcS, CkCS and CkTbS.

**Protein + Substrate**	K_m (μM)	V_{max} (pmol mg^{-1} min^{-1})	k_{cat} (×10^{-3} s^{-1})	k_{cat}/K_m (s^{-1} M^{-1})	R^2
CkTcS + SAM	109.50 ± 25.04	279155.00 ± 21258.00	186.10 ± 14.17	1699.54	0.9794
CkCS + **5**	71.49 ± 15.60	173.40 ± 9.38	0.12 ± 0.01	1.68	0.9749
CkTbS + **5**	162.20 ± 27.80	271.20 ± 9.91	0.18 ± 0.01	1.11	0.9819
CkTcS + **5**	4.68 ± 0.72	17123.00 ± 562.20	11.42 ± 0.37	2440.17	0.9884
CkTcS + **2**	226.20 ± 33.95	9109.00 ± 320.10	6.07 ± 0.21	26.83	0.9881

Supplementary Table 2. Data collection and refinement statistics.

	CkTcS-SAH-1,3,7-trimethyluric acid (6LYH)	CkTbS (6LYI)
**Data collection**		
Space group	P 1	P 6_5
Cell dimensions		
a, *b*, *c* (Å)	87.74,86.81, 123.3	144.8, 144.8, 76.18
α, β, γ (°)	90.21, 90.04, 90.17	90.00, 90.00, 90.00
Resolution (Å)	50.00-3.14(3.20-3.14)[a]	50.00-2.49 (2.53-2.49)
R_{pim}	0.131(0.803)	0.027(0.225)
$I/\sigma(I)$	9.7(2.1)	28.5(2.0)
$CC_{1/2}$	99.5(84.5)	99.9(88.3)
Completeness (%)	96.4(97.8)	99.9(98.4)
Redundancy	2.5(2.4)	19.7(16.3)
**Refinement**		
Resolution (Å)	43.87-3.15	47.39-2.49
No. reflections	55414	34000
R_{work} / R_{free}	0.263/0.298	0.183/0.224
No. atoms		
Protein	20478	5036
Ligand	314	0
Water	0	90
B factors		
Protein	73.0	68.3
Ligand	58.9	-
Water	-	56.0
R.m.s.deviations		
Bond lengths (Å)	0.004	0.006
Bond angles (°)	0.830	1.106

[a]Values in parentheses are for highest-resolution shell.
